# Higher fat mass and fat mass accretion during the first six months of life in exclusively breastfed infants

**DOI:** 10.1038/s41390-019-0542-1

**Published:** 2019-08-21

**Authors:** Ameyalli M Rodríguez-Cano, Jennifer Mier-Cabrera, Ana L Allegre-Dávalos, Cinthya Muñoz-Manrique, Otilia Perichart-Perera

**Affiliations:** Departamento de Nutrición y Bioprogramación, Instituto Nacional de Perinatología “Isidro Espinosa de los Reyes”, Ciudad de México, Mexico

## Abstract

**Background:**

Early nutrition influences infant growth and body composition, which may play a role in the infant’s metabolic programming. Breastfed infants appear to have higher fat mass than formula-fed infants, but most comparisons have been cross-sectional, and evidence is scarce. The aim of this study was to describe fat mass and fat mass accretion during the first six months of life and evaluate differences by type of feeding (OMS).

**Methods:**

Prospective cohort of healthy pregnant women and their infants (Mexico City, 2009–2014). At 1 (T1), 3 (T2) and 6 (T3) months of age, fat mass (FM) (PEAPOD) and type of feeding (feeding questionnaire) were evaluated.

**Results:**

We included 109 healthy infants (mean ± SD age: 39 ± 1.1 weeks; birthweight: 2959 ± 294 g). Exclusive/predominant breastfed (EBF) infants had higher FM at T2 and T3 compared with non-EBF (%FM T3: 29.7 ± 5.9% vs 24.7 ± 5.6%, respectively) (*p* < 0.05). All infants increased their FM throughout time (*p* < 0.001). EBF infants showed a significant higher FM accretion (*β*: 3.61; 95% CI: 1.57–5.66, *p* < 0.01); the difference was maintained after controlling for confounding variables.

**Conclusions:**

Exclusive/predominant breastfeeding promotes higher accretion of FM during the first six months of life which could have an important effect in the programming of health outcomes later in life.

## Introduction

Although global rates of childhood obesity have plateaued, its prevalence remains high, occurring as one of the most important public health problems worldwide.^1^ Intrauterine and early postnatal nutrition have become relevant in the prevention of obesity and other chronic diseases in childhood and adulthood.^2^ Adiposity at birth and during the first months of life is influenced by maternal weight status and the intrauterine environment.^3,4^ Body composition, more than weight-based indices (body mass index, weight-for-length), should be the basis for diagnosing obesity in different age groups. Few studies have assessed body composition in this stage of life.^5–8^ Postnatal nutrition is associated with infant growth and body composition and may play a significant role in metabolic and nutrition programming of the infant.^9,10^

Exclusive breastfeeding is the optimal nutrition recommendation for all infants until 6 months of age. Breastmilk contains all the essential nutrients the baby needs, and provides many bioactive substances and microorganisms with specific properties, that exert different functions in the infant.^11^

Some studies have measured fat mass (FM) and fat-free mass (FFM) at birth and during the first months of life with an air displacement plethysmography equipment,^7,12,13^ a validated method to measure body composition in this age group.^14^ Evidence has shown that breastfed infants appear to have higher FM at 3, 4 and/or 6 months of age. Most studies have shown differences in each time frame, using cross-sectional analysis.^5–8^ One recent study showed that duration of EBF was independently associated with FM percentage at 6 months; breastfeeding (BF) was only associated with subcutaneous fat but not with visceral fat. Higher FM accretion during the first 3 months of age has been associated also with higher FM at 6 months.^8^ In one longitudinal study, a higher increase in FM (as a proportion of body weight) was observed from 3 to 4.5 months of age in exclusive/predominant breastfed infants.^7^ In the beginning of life, higher FM could be part of an optimal phenotype influenced by BF, that may be related to protection in later obesity.^15,16^

The aim of this study was to describe FM and FM accretion during the first six months of life and to evaluate FM differences by type of breastfeeding and other maternal and infant characteristics.

## Methods

### Study settings

This is a secondary analysis derived from a cohort study of healthy pregnant women conducted at the National Institute of Perinatology in Mexico City (INPer; 2009–2014).

### Subjects’ recruitment and selection

Singleton pregnant women (<14 weeks) who did not have comorbidities (diabetes mellitus, autoimmune diseases, or other pathologies) and did not use medication (metformin, prednisone, etc.) during pregnancy that could affect metabolism or body composition were selected consecutively by convenience. Until resolution of pregnancy, women were followed-up monthly. All newborns were followed-up approximately at 1, 3, and 6 months of age.

For this analysis, we included healthy newborns. We did not include newborns from adverse maternal outcomes (gestational diabetes or preeclampsia), with postnatal and/or congenital diseases, preterm birth (<37 weeks), low birthweight (<2500 g), macrosomia (>4000 g), lost to follow-up or that had incomplete feeding information at 6 months.

### Ethical approval and consent to participate

This study was approved by the National Institute of Perinatology’s Institutional Review Board and Ethics Committee (reference number: 212250-49511). All procedures were conducted according to the Declaration of Helsinki. We obtained signed informed consent from all participants; in adolescents (<19 years old) both parents and participants gave consent.

### Maternal data collection

Pregestational maternal weight was self-reported. Height was measured using a wall-mounted wireless digital stadiometer SECA 242 (SECA, Hamburg, Germany). Pregestational BMI (preBMI) was computed and women were classified as low weight, normal weight, overweight, or obesity according to the World Health Organization (WHO) criteria.^17^ Taking into consideration women’s preBMI, gestational weight gain (GWG) in the third trimester (last visit recorded) was classified as low, adequate, or high according to the Institute of Medicine (IOM) recommendations.^18^ Parity was classified as either nulliparous or primiparous/multiparous. Women were classified in the following categories regarding level of education: basic (elementary and middle school), middle (high school and/or technical level) or high (bachelor and postgraduate).

### Infant data collection, anthropometry, and body composition

Gestational age (weeks) was estimated by ultrasound during the first trimester. In cases where no ultrasound was available, gestational age was calculated according to woman’s last menstrual period.

Infants were measured at birth (first 72 h) and the follow-up consisted in three visits around 1 (T1), 3 (T2) and 6 (T3) months of age. While infants were without clothes, weight, and length were measured by an experienced and trained nutritionist using a standardize technique proposed by Lohman et al.^19^ Weight at birth was recorded using a pediatric scale 1582 Baby/Mommy Scale (Tanita, Tokyo, Japan) whereas for subsequent visits, the PEAPOD Infant Body Composition System digital scale (COSMED USA Inc, Concord, California) was used. Recumbent length was measured (by duplicate and the average computed) using an infantometer SECA 207 (SECA, Hamburg, Germany).

Nutritional status was assessed using the WHO reference data^20^ for BMI-for-age (BMI/A). Sex-specific *z*-scores were calculated using the Anthro software v. 3.2.2 (WHO, Geneva, Switzerland). Risk of overweight, overweight, and obesity was defined by means of BMI/A (>1, >2 and >3 *z*-score, respectively), while a *z*-score < −2 was considered as wasted/severely wasted.

FM (percentage—%FM and kilograms—kgFM) was measured at T1, T2, and T3 using the PEAPOD, an air displacement plethysmography system. Before starting the measurements, the PEAPOD was calibrated following the manufacturer’s protocol. First, the infant was naked and was weighed using the PEAPOD scale. Then, to minimize air trapped in the hair, a cap was put on his/her head. Thereafter, the infant was placed in the PEAPOD’s test chamber tray to begin the measurement of body composition.^14,21^ The whole process took about 5 min. Finally, based on Fomon’s density values, the PEAPOD’s software calculated the infant’s body composition. FM index (FMI) was calculated dividing kgFM/length^2^ to account for body size natural variations.^22^

### Feeding practices questionnaire

In every visit, except at birth, mothers were asked about their infant feeding practices. The main purpose was to evaluate whether infants were breastfed, formula-fed or both, as well as if they had ever been fed any other food/liquid (e.g. water, juices, infusions, other types of milk, semi-solid or solid food) during the first six months of life. Duration of BF was recorded in months. Taking into account WHO definition of breastfeeding,^23^ infants were classified as exclusive/predominant breastfed (EBF), when lactation lasted 6 months, or non-exclusive/predominant breastfed (nEBF), which included mixed feeding and formula feeding. Complementary feeding (CF) was recorded as the age (in months) when an infant received solid/semi-solid food different from breastmilk, infant formula, water or infusions for the first time. CF was classified as “early” if it started <4 months of age.

### Statistical analysis

Descriptive statistics and frequencies were performed for all variables. Mean differences were analyzed using *t* test/*U* Mann–Whitney or ANOVA/Kruskal–Wallis tests. Chi-square test was used to analyze differences between infant and maternal categories. Pearson’s and Spearman’s correlations were used to evaluate bivariate associations.

Generalized linear mixed models (GLMM) were used to evaluate the association between EBF and FM (%, kg, FMI) during the first six months of life. According to the literature and based on our exploratory analysis, three GLMM were performed. The first GLMM did not include confounding variables; the second, included variables that were associated with FM in the exploratory analysis (parity, level of education, and category of GWG). The third model included the same variables of the second model (parity, level of education, and category of GWG) and independent variables that have been reported to influence FM (gender, gestational age at birth, BMI/A at birth, start of CF).

A *p*-value < 0.05 was considered statistically significant. Statistical analyses were performed with SPSS Statistics Software v.24 (IBM Corp, Armonk, New York). GLMM were performed with Stata statistical software package v.12 (StataCorp, College Station, Texas).

## Results

### Participants

**Fig. 1:**
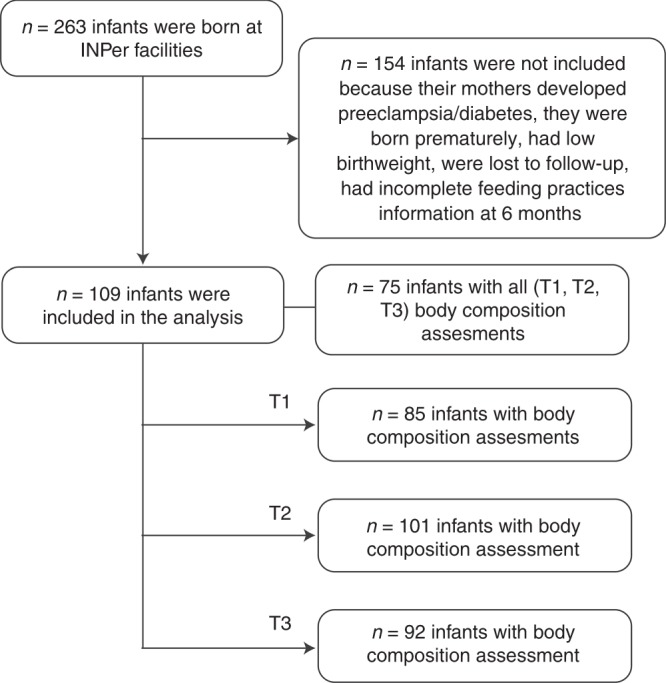
Flow chart of participants

A total of 263 babies from the cohort were born at INPer facilities. One hundred and fifty-four newborns were not included in this analysis because their mothers developed preeclampsia (*n* = 5) or gestational diabetes (*n* = 6), they were born prematurely (*n* = 30), had low birthweight (*n* = 13), were lost to follow-up (*n* = 67) or had incomplete feeding practices information available at 6 months (*n* = 33). Finally, data from 109 infants were analyzed in this study (Fig. 1).

### Maternal information

Mothers’ mean age was 27.1 ± 8.9 years; 23% (*n* = 25) of them were adolescents and 58.7% (*n* = 64) were nulliparous. Their mean preBMI was 25.3 ± 5.4 kg/m^2^ and the prevalence of pregestational overweight and obesity was 27.5% (*n* = 30) and 15.6% (*n* = 17), respectively (Table 1). GWG was low in 30.9% (*n* = 30) and excessive in 47.4% (*n* = 46) of women. Most of them were classified in the low or middle category regarding level of education (75.5%).

**Table 1:** Descriptive data of mothers and infants according to type of feeding

	EBF			nEBF		
	All	Girls	Boys	All	Girls	Boys
Maternal information	*n* = 28	*n* = 10 (35.7)	*n* = 18 (64.3)	*n* = 81	*n* = 41 (50.6)	*n* = 40 (49.4)
Age (years)	28.0 (21.0–35.3)	23.5 (17.8–34.0)	30.0 (23.3–36.3)	26.0 (16.5–35.0)	23.0 (16.0–33.0)	30.5 (19.3–37.5)
Adolescents	3 (10.7)	2 (20.0)	1 (5.6)	22 (27.2)	14 (34.1)	8 (20.0)
Adults	25 (89.3)	8 (80.0)	17 (94.4)	59 (72.8)	27 (65.9)	32 (80.0)
Pregestational weight (kg)	58.0 (54.0–63.9)	57.0 (50.5–64.5)	58.5 (54.3–65.4)	61.0 (53.5–69.5)	59.0 (50.0–69.5)	62.0 (55.0–71.3)
PreBMI (kg/m^2^)	23.5 (21.726.8)	24.4 (21.3–27.1)	23.4 (21.7–27.2)	24.3 (21.5-28.6)	24.0 (20.9–27.4)	25.5 (22.6–28.8)
Low weight	0 (0.0)	0 (0.0)	0 (0.0)	2 (2.5)	2 (4.8)	0 (0.0)
Normal weight	17 (60.7)	5 (50.0)	12 (66.7)	43 (53.1)	25 (61.0)	18 (45.0)
Overweight	8 (28.6)	5 (50.0)	3 (16.7)	22 (27.2)	7 (17.1)	15 (37.5)
Obesity	3 (10.7)	0 (0.0)	3 (16.7)	14 (17.2)	7 (17.1)	7 (17.5)
3th trimester GWG categories						
Low	7 (29.2)	1 (11.1)	6 (40.0)	23 (31.5)	15 (39.4)	8 (22.9)
Adequate	9 (37.5)	4 (44.4)	5 (33.3)	12 (16.4)	5 (13.2)	7 (20.0)
Excessive	8 (33.3)	4 (44.4)	4 (26.7)	38 (52.1)	18 (47.4)	20 (57.1)
Parity categories						
Nulliparous	13 (48.1)	6 (60.0)	7 (41.2)	51 (68.0)	26 (70.3)	25 (65.8)
Primi/Multiparous	14 (51.9)	4 (40.0)	10 (58.8)	24 (32.0)	11 (29.7)	13 (34.2)
Level of education categories						
Basic	11 (40.7)	6 (60.0)	5 (29.4)	24 (32.0)	13 (35.1)	11 (28.9)
Middle	13 (48.1)	3 (30.0)	10 (58.8)	29 (38.7)	14 (37.8)	15 (39.5)
High	3 (11.1)	1 (10.0)	2 (11.8)	22 (29.3)	10 (27.0)	12 (31.6)
Infants information						
Gestational age (weeks)	38.5 (38–39.5)	38.6 (37.9–40.2)	38.5 (37.9–39.3)	39.1 (38.4–40)	39.3 (38.5–40.0)	39.0 (38.3–40.1)
37.0–38.6 weeks	17 (60.7)	6 (60.0)	11 (61.1)	30 (37.0)	12 (29.3)	18 (45.0)
>39 weeks	11 (39.3)	4 (40.0)	7 (38.9)	51 (63.0)	29 (70.7)	22 (55.0)
Birth weight (g)	2960 (2755–3209)	2930 (2635–3130)	2960 (2768–3330)	2960 (2732.8–3120)	2810 (2705–3080)	3000 (2743–3225)
Birth length (cm)	47.4 ± 1.6	46.5 ± 1.2	47.8 ± 1.7	47.1 ± 1.8	46.8 ± 1.8	47.4 ± 1.9
Birth BMI/A	13.3 ± 0.9	13.6 ± 1.2	13.2 ± 0.8	13.3 ± 0.9	13.3 ± 0.8	13.2 ± 1.1
Start of CF (month)	5.0 (4.0–6.0)^*^	4.0 (3.8–5.3)	5.0 (3.8–6.0)^§^	4.0 (3.0–5.0)^*^	4.0 (3.0–5.0)	4.0 (3.0–5.0)^§^
<4 months	6 (21.4)	2 (20.0)	4 (22.2)	22 (28.6)	13 (32.5)	9 (24.3)
>4 months	22 (78.6)	8 (80.0)	14 (77.8)	55 (71.4)	27 (67.5)	28 (74.7)

Data is showed either as Mean ± SD; Median (25° percentile–75° percentile); *n* (percentage)

*U* Mann–Whitney ^*^p = 0.023, All (EBF vs nEBF); ^**§**^*p* = 0.033, Boys (EBF vs nEBF)

*EBF* exclusive/predominant breastfeeding, *nEBF* non-exclusive/predominant breastfeeding, *PreBMI* pregestational BMI, *GWG* gestational weight gain, *BMI/A* Body-mass-index/age, *CF* complementary feeding

### Feeding practices

Mean duration of BF was 5.0 ± 1.9 months. Throughout their first six months of life, only 25.7% (*n* = 28) of the infants were EBF. Infants from women with obesity did not show a lower EBF exposure or duration of BF; there was no linear association between preBMI and duration of BF. Duration of BF and EBF exposure were not different by GWG categories.

Boys and girls had similar EBF exposure and duration of BF. Infants from women classified as nulliparous or in the high level of education category did not show a higher EBF exposure or BF duration. Infants started CF at a mean age of 4.1 ± 1.3 months and early CF was observed in 25.7% (*n* = 28) of them. nEBF infants started CF earlier (*p* = 0.023) (Table 1).

### Anthropometry and body composition

Mean gestational age at birth was 39.0 ± 1.1 weeks. Newborns weighed 2959 ± 294 g and were 47.2 ± 1.8 cm long. Median age (min–max) at T1, T2, and T3 was 4.9 (3.6–8.7), 13.4 (11–17.4) and 26.4 (21.3–38.1) weeks, respectively. Boys had a higher weight throughout the study (except at birth) and higher length in all visits (except at T1) (*p* < 0.05). Most of them (93.8%, *n* = 91) had a normal BMI/A at birth. Although no infants were classified with obesity during the first six months of life, overweight reached its highest prevalence at T3 (5.0%, *n* = 5).

Table 2 shows the increase of FM throughout time (T1, T2, and T3) according to feeding practices. Respectively, boys’ and girls’ mean %FM were as follows: T1: 16.3 ± 4.9%, 17.3 ± 5.4%; T2: 24.9 ± 5.1%, 24.4 ± 5.3% and T3: 25.5 ± 5.5%, 25.9 ± 6.3%.

**Table 2:** Body composition and anthropometric data according to type of feeding

	EBF			nEBF		
	All	Girls	Boys	All	Girls	Boys
T1 [4.9 (3.6–8.7) weeks]	*n* = 18	*n* = 5	*n* = 13	*n* = 67	*n* = 33	*n* = 34
Body composition						
kgFM	0.81 ± 0.31	0.97 ± 0.39	0.74 ± 0.26	0.70 ± 0.27	0.70 ± 0.24	0.71 ± 0.30
%FM	19.2 (15.3–22.4)	22.9 (16.0-27.5)	18.9 (13.4–21.8)	17.9 (12.2–20.9)	18.3 (12.3–21.0)	16.7 (12.0–20.9)
FMI (kgFM/length^2^)	2.9 ± 1.1	3.6 ± 1.4^§^	2.7 ± 1.0	2.5 ± 0.9	2.6 ± 0.8^§^	2.5 ± 0.9
Anthropometry						
Weight (g)	4270 ± 550	4313 ± 735	4255 ± 502	4115 ± 504	3992 ± 406	4249 ± 568
Length (cm)	52.5 ± 1.8	51.9 ± 2.1	52.7 ± 1.7	52.3 ± 2.2	51.9 ± 2.2	52.6 ± 2.1
BMI	15.5 ± 1.8	16.0 ± 2.3	15.3 ± 1.4	15.0 ± 1.1	15.5 ± 1.8	15.5 ± 1.8
T2 [13.4 (11.0–17.4) weeks	*n* = 27	*n* = 10	*n* = 17	*n* = 74	*n* = 36	*n* = 38
Body composition						
kgFM	1.64 ± 0.51^*^	1.74 ± 0.54^§§^	1.57 ± 0.49	1.42 ± 0.40^*^	1.30 ± 0.35^†, §§^	1.53 ± 0.41^†^
%FM	27.1 ± 5.7^**^	28.9 ± 5.3^§§^	26.0 ± 5.8	24.0 ± 4.8^**^	23.5 ± 4.7^§§^	24.4 ± 4.9
FMI (kgFM/length^2^)	4.7 ± 1.3^*^	5.1 ± 1.3^§§^	4.5 ± 1.3	4.1 ± 1.1^*^	3.9 ± 1.0^§§^	4.3 ± 1.1
%FM T2 – %FM T1	8.9 ± 5.7	9.7 ± 5.8	8.6 ± 5.9	7.3 ± 4.4	6.0 ± 3.9^†^	8.5 ± 4.5^†^
Anthropometry						
Weight (g)	5973 ± 803	5794 ± 839	6017 ± 803	5808 ± 718	5475 ± 614	6142 ± 664
Length (cm)	58.6 ± 2.0	57.9 ± 2.0	59.0 ± 2.0	58.4 ± 2.1	57.4 ± 2.1	59.3 ± 1.7
BMI	17.3 ± 1.5	17.5 ± 1.5	17.2 ± 1.6	17.0 ± 1.5	16.6 ± 1.4	17.4 ± 1.6
T3 [26.4 (21.3–38.1) weeks]	*n* = 20	*n* = 8	*n* = 12	*n* = 72	*n* = 38	*n* = 34
Body composition						
kgFM	2.22 ± 0.67^**^	2.54 ± 0.76^§§§^	2.00 ± 0.54	1.79 ± 0.53^**^	1.69 ± 0.48^§§§^	1.91 ± 0.57
%FM	29.7 ± 5.9^**^	32.5 ± 6.3^§§^	27.9 ± 5.1	24.7 ± 5.6^**^	24.5 ± 5.6^§§^	24.9 ± 5.7
FMI (kgFM/length^2^)	5.4 ± 1.6^**^	6.3 ± 1.7^‡, §§§^	4.8 ± 1.2^‡^	4.3 ± 1.2^**^	4.2 ± 1.1^§§§^	4.5 ± 1.3
%FM T3 – %FM T1	11.5 (6.6–14.6)	11.5 (5.0–17.3)	11.6 (6.6–14.6)	7.3 (5.1–11.1)	7.2 (3.8–10.9)	7.6 (5.9–11.5)
%FM T3 – %FM T2	2.2 ± 2.7	2.9 ± 2.3	1.8 ± 2.9	1.3 ± 3.8	1.6 ± 3.6	1.0 ± 4.0
Anthropometry						
Weight (g)	7518 ± 988	7675 ± 1018	7424 ± 992	7197 ± 868	6866 ± 70	7545 ± 895
Length (cm)	64.6 ± 2.4	63.8 ± 2.7	65.1 ± 2.2	64.3 ± 2.6	63.4 ± 2.2	65.3 ± 2.7
BMI	18.0 ± 2.0	18.8 ± 2.1	17.5 ± 1.7	17.4 ± 1.5	17.1 ± 1.3	17.7 ± 1.7

Data is showed either as Mean ± SD. Median (25° percentile–75° percentile)

*t* test: EBF vs nEBF, ^*^p < 0.05, ^**^p < 0.01; EBF (girls vs boys), ^‡^*p* = 0.0034; nEBF (girls vs boys), ^†^*p* < 0.05; girls (EBF vs nEBF), ^§^*p* < 0.05, ^§§^*p* < 0.01, ^§§§^*p* < 0.001

*EBF* exclusive/predominant breastfeeding, *nEBF* non-exclusive/predominant breastfeeding, *T1/T2/T3 Median (Min–Max) weeks* time frame at each assessment, *kgFM* kilograms of fat mass, *%FM* fat mass percentage, *FMI* fat mass index, *BMI* body-mass-index

FM was not different according to preBMI, gender, BMI/A at birth, or gestational age in any visit. At T3, FMI was higher in babies from mothers in the basic level of education category in comparison to those in the middle category (*p* = 0.029). At T2 and T3, those infants from mothers with low GWG showed lower FM when compared with those with adequate (T2: %FM *p* = 0.001; kgFM *p* = 0.002; FMI *p* = 0.001; T3: %FM *p* = 0.008; kgFM *p* = 0.012; FMI *p* = 0.003) or excessive GWG (T2: %FM *p* = 0.003, kgFM *p* = 0.001; FMI *p* = 0.003; T3: kgFM *p* = 0.042). A higher FM (%FM *p* = 0.049; kgFM *p* = 0.027; FMI *p* = 0.036) at T2 was observed in infants born from primiparous/multiparous mothers.

EBF infants had higher FM at T2 (%FM, *p* = 0.008; kgFM *p* = 0.027; FMI, *p* = 0.021) and T3 (%FM, *p* < 0.001; kgFM, *p* = 0.004; FMI, *p* = 0.001) compared with nEBF ones (Table 2). Positive correlations were found between duration of BF and FM (kgFM: *r* = 0.234, *p* = 0.018; FMI: *r* = 0.200, *p* = 0.045) at T2. Infants with early CF did not show differences in FM (%FM, kgFM, FMI). FM did not correlate with starting of CF.

### General linear mixed models: type of breastfeeding and FM

**Fig. 2:**
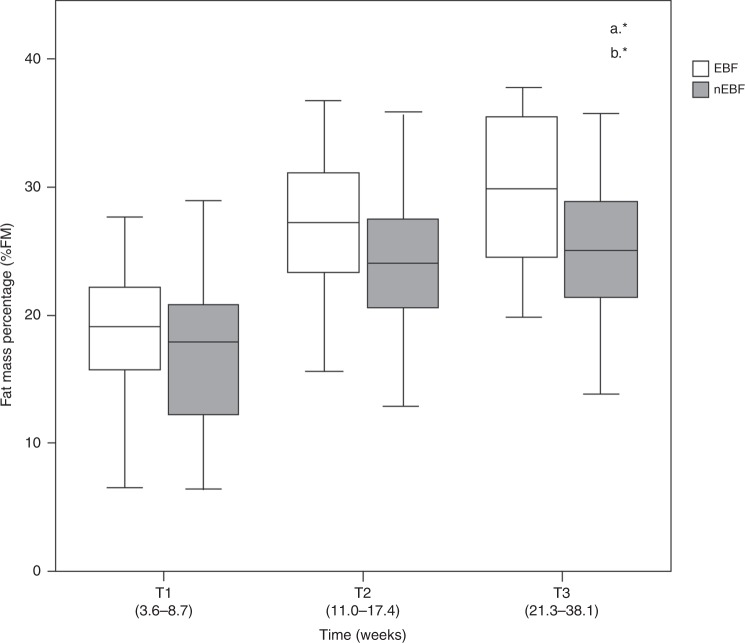
Fat mass percentage during the study according to type of feeding

All infants increased their FM (%, kg, and FMI) significantly throughout time (*p* < 0.001; a*) (Fig. 2). The greatest increase in FM was observed from T1 to T2 (kg [*β* = 0.72 (95% CI 0.63, 0.82)]; % [*β* = 7.42 (95% CI 6.35, 8.48)]. EBF infants showed a significant higher accretion in FM (%, kg, and FMI) when compared with those nEBF (*p* < 0.01; b*) (Fig. 2). This difference was maintained after controlling for maternal and infant variables in the three models (*p* < 0.01). None of the confounding variables or co-variables showed a significant effect on FM, except for GWG in the second model; infants from women with excessive GWG, compared with those with low GWG, had a higher increase of FM (kg [*β* = 0.25 (95% CI 0.06, 0.45) *p* = 0.008]; % [*β* = 2.79 (95% CI 0.53, 5.05) *p* = 0.015]) and FMI [*β* = 0.62 (95% CI 0.11, 1.12) *p* = 0.016] (Table 3).

**Table 3:** Effect of exclusive breastfeeding on fat mass increase from T1 to T3

	kgFM		%FM		FMI	
	*β* (95% CI)	*p*	*β* (95% CI)	*p*	*β* (95% CI)	*p*
Model 1^a^						
EBF^e^	0.24 (0.06–0.41)	0.008	3.29 (1.26–5.32)	0.001	0.65 (0.20–1.10)	0.005
T2^f^	0.74 (0.65–0.82)	0.0001	7.53 (6.56–8.51)	0.0001	1.63 (1.43–1.84)	0.0001
T3	1.17 (1.09–1.26)	0.0001	8.84 (7.85–9.84)	0.0001	1.97 (1.76–2.17)	0.0001
Model 2^b^						
EBF	0.30 (0.10–0.50)	0.003	3.83 (1.48–6.18)	0.001	0.80 (0.28–1.32)	0.003
T2	0.73 (0.64–0.82)	0.0001	7.42 (6.36–8.49)	0.0001	1.60 (1.38–1.83)	0.0001
T3	1.16 (1.07–1.24)	0.0001	8.74 (7.66–9.82)	0.0001	1.95 (1.72–2.18)	0.0001
Model 3^c^						
EBF	0.42 (0.17–0.67)	0.001	5.16 (2.18–8.14)	0.001	1.12 (0.46–1.78)	0.001
T2	0.73 (0.62–0.83)	0.0001	7.42 (6.24–8.60)	0.0001	1.60 (1.34–1.85)	0.0001
T3	1.15 (1.04–1.26)	0.0001	8.50 (7.31–9.69)	0.0001	1.90 (1.64-2.15)	0.0001

No significant association was observed with other variables besides EBF and Time in the 3 models and GWG only in Model 2

*kgFM* kilograms of fat mass; *%FM* fat mass percentage; *FMI* Fat mass index; *EBF* exclusive/predominant breastfeeding; *T2/T3* Time frame

^a^Crude GLMM (type of feeding^e^ -when compared with nEBF- and T2/T3^f^ -when compared with T1-)

^b^Model 1 + adjustment by parity, maternal level of education and category of gestational weight gain (GWG) (when compared to low GWG)

^c^Model 2 + adjustment by gestational age at birth, gender, BMI/A at birth (*z*-score), start of complementary feeding (month)

## Discussion

This study presents evidence about the influence of BF on body composition in healthy term infants during the first six months of life, where FM accretion was higher in EBF infants. We also observed higher FM from week 11 to week 38.1 of age in EBF infants.

Our results are in line with previously reported findings where differences in FM have been observed, either at a specific time or in regard to its increase throughout time. In our analysis, we found differences in both scenarios. Butte et al.^5^ found a higher FM (kg and %) at 3 and 6 months in EBF infants vs formula-fed (FF) ones, although FM was evaluated with different methodologies (TOBEC, multicomponent body composition model). Carberry et al.^7^ is the only previous longitudinal study that found a higher increase in FM as a proportion of weight gain in EBF infants, but the time frame studied was very short (from 3 to 4.5 months). Our analysis showed FM accretion (kg, %, FMI) over an 8-month time frame.

Some other authors have found discrepancies. In a descriptive study by Gianni et al.,^6^ no differences in FM according to type of feeding were observed, but a higher FFM was reported in FF infants. Probably, these different outcomes were as a result of their hypothesis focused on finding differences in FFM (the power proposed was not enough to find differences in FM) and also women with a preBMI > 25 were excluded. Inconsistent findings in the literature could be as a result of methodologic differences, such as sample size, inclusion/exclusion criteria, time frame, and methods for anthropometric/body composition assessment, statistical analysis, etc.

Breij et al.^12^ assessed %FM and visceral fat at 1, 3 and 6 months. Duration of EBF was positively associated with %FM (PEAPOD) at 6 months, but not with visceral fat (ultrasound). This finding is of particular interest because location of body fat storages determines metabolic risk.^24^

Our results showed that EBF appears to increase FM accretion during the first six months of life. This may be a desirable outcome that could be supporting other aspects of healthy growth (e.g. ensure infant’s normal brain development).^25^ Leptin and adiponectin, present in human milk, have important roles on energy homeostasis, satiety regulation, immune system, glycemic control, among other functions that are related to growth and changes in body composition.^26,27^ Brunner et al.^26^ found that milk leptin is inversely associated with FFM, and adiponectin could be directly associated to infant FM (based on skinfolds) at 4 months of age. In addition, Breij et al.^12^ found a positive correlation between infant serum leptin and %FM at 3 months of age. There is a strong need to understand how these substances modulate growth, body composition, and long-term health.

As previously reported, FM has its higher increase during the first 4 months of life,^9,28^ and thereafter accretion velocity appears to decrease. Our model showed this rapid increase from week 3.6 to week 17.4 of age, and an attenuation from week 17.4 to week 21.3 of age. We hypothesize that in EBF infants, FM could continue with this slow accretion and result in a lower FM (and lower obesity risk), noticeable by 12 months of age, when compared with those infants with previous formula exposure. Interestingly, Gale et al. found that higher FM at 3 and 6 months tends to invert at 12 months of age, showing higher FM in FF infants when compared with those EBF.^29^ Studies in older children have shown that greater duration of BF was associated with a lower FM at 4^30^ and at 9–10 years of age.^31^ Furthermore, as opposite to EBF infants, those exposed to formula have higher protein intake^5^ and may be introduced to solid foods earlier;^31^ both practices have been related to higher adiposity and associated disorders.^32,33^ This could reflect the long-term protective effect of breastmilk, showing the lower risk of obesity in childhood.^16^ This panorama emphasizes the importance of studying changes and distribution of FM since birth, as well as the need of FM reference values in order to better assess nutrition status and metabolic risks in infants.

Besides feeding type, there are other factors known to influence FM in infants. Our results showed FM differences according to category of GWG. GWG is correlated with preBMI, and both have been related to adiposity in the newborn^3,34^ and with a higher long-term obesity risk.^35,36^ However, the association between excessive GWG and FM disappeared when other infant variables were considered, suggesting an intermediary role between fetal programming and the extra uterine environment. Extremes of BMI/A at birth may have an influence on susceptibility to later obesity. Our results did not show any influence of BMI/A at birth on FM during the first six months of life. This could be related to the fact that most infants had normal weight, and there were no low weight or obese infants. In addition, early CF (<4 months) could increase the risk of childhood overweight (classified by BMI), but data is inconclusive.^32^ We did not find that FM was influenced by an early start of CF.

There is also conflicting evidence about differences in FM by gender. Although there is some data that females have higher FM compared with males, some authors had not found differences at birth or very early in life.^7,37^ Our reported %FM is very similar to what has been previously reported.^38,39^

Association between BF and obesity may be affected by residual confounding, especially as a result of mothers’ socioeconomic status and level of education. Higher education and income has been associated with higher frequency and duration of BF.^5,16^ We found that a lower level of education was associated with higher FMI at T3; however, this effect was not significant in the GLMM. Durmusx et al.^40^ found that when associating BF and FM, maternal level of education was the strongest confounder, leading to a non-significant association. Others have found that the duration of BF has a significant effect in FM, even after adjusting for maternal characteristics.^30,31^

This study has some strengths. This is one of the few studies that have assessed BF and FM prospectively during the first six months of life. FM was measured using a validated method in infants. Changes in FM are rapid and nonlinear in the first months of life;^37^ therefore, longitudinal studies are needed during this time frame. One of the most common critiques of BF studies is their lack of control for confounders (maternal and infant variables, ethnicity, socioeconomic status, etc.).^27^ We included variables that are known to influence adiposity (parity, level of education, BMI/A at birth, gender, gestational age, and CF), although neither showed statistical significance. Finally, our sample comes from a low-/middle-income population from a developing country where evidence is scarce, as most of the studies have been reported from high-income countries.^16^

This analysis presents some limitations. Weeks of FM measurements were not the same for all infants. Although the variability in the time frame within the three FM measurement periods was high, when we adjusted the model including “time” as a continuous variable (weeks), the effect of EBF on FM was maintained (data not shown). Feeding type classification is complex as it relies on the mother’s report and is subject to re-call bias. Women in our study are not representative of all women because they may have high-risk factors as they were selected from a tertiary referral hospital.

Exclusive/predominant breastfeeding promotes higher accretion of fat mass during the first six months of life which could have an important effect in the programming of health outcomes later in life. Accretion of fat mass is higher in the first 3 months of life, representing a critical period of growth. More longitudinal studies are needed to explain the effect of EBF on fat mass at early age and the implication of this on nutrition status and later metabolic risk.
